# Disordered eating behaviours and body shape dissatisfaction among adolescents with type 1 diabetes: a cross sectional study

**DOI:** 10.1186/s40337-023-00876-y

**Published:** 2023-09-26

**Authors:** Lidiya Daniel, Demewoz Haile, Gudina Egata

**Affiliations:** Department of Nutrition and Dietetics, School of Public Health, College of Health Sciences, Addis Ababa University, Addis Ababa City, Ethiopia

**Keywords:** Diabetes eating problem survey-revised (DEPS-R), Binge eating, Disordered eating behaviour, Body shape dissatisfaction, Adolescents, Type 1 diabetes, Purging, Ethiopia

## Abstract

**Background:**

Disordered eating behaviours (DEBs) are variations in regular eating patterns and behaviours and might include symptoms and behaviours of eating disorder with lower level of severity. Such behaviours are common during adolescence at which time several physical and psychological changes occur favouring unhealthy dietary behaviours. Although the magnitude of DEBs is high among high—income countries, similar data are limited among adolescents with diabetes in low-income countries including Ethiopia. This study aimed to assess the magnitude of DEBs and its relationship with body shape dissatisfaction among adolescents with diabetes on follow-up at selected public hospitals in Addis Ababa, Ethiopia.

**Methods:**

Hospital based cross sectional study was conducted among randomly selected 395 adolescents with diabetes attending public hospitals in Addis Ababa from January to December, 2021. Data were collected using structured pretested standard diabetes eating problem survey revised (DEPS-R) questionnaire, body part satisfaction scale of 8 items, and anthropometric measurements. Descriptive statistics such as median alongside interquartile range was used to describe the continuous variables. Binary bivariable and multivariable logistic regression was used for data analysis. Mann–Whitney U-test and Kruskal–Wallis test were used to evaluate the difference between median scores of independent variables. Adjusted odds ratios (AOR) alongside 95% confidence intervals (CIs) were estimated to measure the strength of association between variables of interest.

**Results:**

The magnitude of disordered eating behaviours within the last 30 days was 43.3%, [95% CI: (38%, 48%)]. In multivariable analysis, body shape dissatisfaction [AOR = 2.21, 95% CI (1.28, 3.82, *p* = 0.0001)], family history of diabetes mellitus [AOR = 1.59, 95% CI (1.03, 2.47, *p* = 0.038)], late adolescence period [AOR = 2.10, 95% CI (1.33, 3.34, *p* = 0.002)], having diabetic complication[AOR = 2.32, 95% CI (1.43, 3.75, *p* = 0.001)],and being overweight [AOR = 2.25, 95% CI (1.32, 3.82, *p* = 0.003)] were significantly associated with DEBs.

**Conclusions:**

The magnitude of DEBs was high among the study participants. Body shape dissatisfaction, family history of diabetes mellitus, being in late adolescence period, diabetic complication, and nutritional status of adolescents were significantly associated with DEBs. Therefore, preventive interventions need to be designed by all relevant actors working on health promotion of young population to address factors influencing DEBs among adolescent population with diabetes.

## Abstract (plain language summary)

The World Health Organization (WHO) defined adolescents as individuals in the age bracket of 10–19 years. Biologically, adolescence is a period of development that stretches from the onset of puberty through the termination of growth. This period is a critical link between childhood and adulthood, characterized by significant physical, psychological, and social transitions which carry new risks including the development of disordered eating behaviors among adolescents and opportunities that influence the immediate and future health of young people. This cross-sectional study aimed at assessing the magnitude of disordered eating behaviors and its relationship with body shape dissatisfaction among adolescents with diabetes on follow-up at selected public health facilities. The results showed that the magnitude of disordered eating behaviors is high. Body shape dissatisfaction was found to be statistically significantly associated with disorder eating behaviors during adjusted analysis alongside other attributes which have been identified to have an influence on adolescents’ eating behaviours. A better understanding of the link between individual level attributes and DEBs could help adolescents’ health programmers to launch more conducive interventions targeting the identified risks and researchers to more understand other aspects that could influence the adolescents’ eating behaviors.

## Background

Adolescence is a critical period of human development during which several physical and psychological changes occur, and unfortunately, some of them can favour unhealthy dietary behaviours due to a transition from childhood dependency to adult self-sufficiency [1]. Adolescents are the adults of tomorrow, and the quality and quantity of food they eat determine their growth, progress, and potential health consequences for the rest of their lives [2].

Disordered eating behaviours (DEBs) are serious and under-recognized problems in people with diabetes and are associated with poor health outcomes [3]. DEBs are variations in regular eating patterns and behaviours which may include less severe symptoms and behaviours of eating disorders and a variety of compensatory mechanisms such as dieting, long hour fasting (≥ 24 h), skipping meal, binge-eating, using diet pills and other purgative behaviours like self-induced vomiting, using diuretics or laxatives and tend to rise among adolescent population [4].

Globally, former studies from wealthier countries affirmed that DEBs are known to be more prevalent ranging from 8.69% to 59% [5–13] among adolescents with type 1 diabetes mellitus (T1-DM). On the other hand, available evidence from Africa, other than Ethiopia, indicated that the prevalence of DEBs ranges from 10 to 34.7% [14–16].

DEBs are harmful to patients with diabetes, especially those who are on insulin [17]. These patients are at risk of bad glycemic function which can be related to a number of health conditions, including macro vascular (heart disease) and micro vascular (eye, kidney, and nerve damage) complications [18]. Accordingly, evidence indicated that individuals with severe DEBS had nearly three-fold some degree of retinopathy and nephropathy compared to those who had no any disordered eating [19]. Disordered eating can also lead to reduced psychological functioning and reduced quality of life [3].

Different studies revealed that DEBs are linked to socio-demographic characteristics such as gender [3, 20, 21] and age [10, 22, 23], nutritional status, having a higher body mass index for age (BAZ) score [10, 21, 22, 24, 25] and clinical constructs including a higher HbA1c [10, 22], higher level of total cholesterol and low density lipoprotein (LDL) [10],depression and anxiety [16], and insulin restriction [22, 26] among others. Accordingly, DEBs were found to be more common among adolescents aged 17–19 years affecting up to 50% of adolescents aged 18 years [10, 22]. Girls are more affected than boys (34.1% and 8.9%) respectively. A multi-country cross-sectional analysis in the Caribbean region also confirmed that females are more affected by DEBs [3, 20, 21] and unhappy with their shape and bodies than boys [21]. A number of recent studies revealed bi-directional relationships between disordered eating and depression and anxiety symptoms and quality of life in populations with T1-DM and type 2 diabetes mellitus (T2-DM) [3, 16]. Evidence also indicated that weight loss related with T1-DM induction, weight increase following diagnosis and beginning of intensive insulin treatment, and body shape dissatisfaction (BSD) are all factors to consider as predictors of DEBs [27].

Body shape dissatisfaction (BSD), a person's negative perceptions, feelings, and thoughts about his or her body, is experienced by around 32% of adolescent females and 14.6% of adolescent males [28]. Some studies had shown an association between body weight dissatisfaction and variables such as unhealthy eating behaviours and lack of physical activity during adolescence. BSD is more common among girls than boys and overweight adolescents than underweight adolescents [22, 28–31].

Preventive efforts aiming at reducing negative risk factors like BSD, depression, low self-esteem on appearance and increasing protective factors such as a non-appearance-oriented self-definition and replacing dieting and body dissatisfaction with intuitive eating and appreciation for the body’s functionality have been developed and evaluated all over the world particularly in better-off nations [32]. Nevertheless, there are no strategies and programs concerning disordered eating attitude among Ethiopian adolescents.

Despite the fact that many studies have been conducted in developed countries to investigate the association between DEBs and BSD, there is scarcity of evidence in low-income countries like Ethiopia. Available evidence from Ethiopia that assessed unhealthy weight control behaviors and disordered eating attitudes and associated factors among Ethiopian healthy female adolescents reported that a significant number of adolescents were susceptible to developing disordered eating attitude [33, 34]. Hence, it is very important to understand the magnitude of DEBs and its association with BSD and other correlates among adolescents with diabetes to design crucial public health programs and interventions in the context of the study setting in particular and in Ethiopia in general. Therefore, this study aimed to assess the magnitude of DEBs and its relationship with BSD among adolescents with diabetes, in Addis Ababa, Ethiopia.

## Methods

### Study setting, period, design, and population

Hospital based cross-sectional study was conducted among adolescents with diabetes aged (10–19 years) on follow-up at diabetes center of five public hospitals namely, Black Lion specialized hospital, Yekatit 12 hospital and medical college, St Paul specialized hospital, Zewditu memorial and Menelik II referral hospital) in Addis Ababa, Ethiopia from September to November, 2021. Addis Ababa is a capital city of federal democratic republic of Ethiopia with a population of 3,384,569 [35]. Currently, the city has 12 state run hospitals most of which have diabetes treatment clinics with varying patient flow. There were a total of 2637 adolescents aged 10–19 years with T1-DM who were on follow-up for one year in selected hospitals. All randomly selected adolescents aged 10–19 years with T1-DM who were on follow- up and reported insulin therapy were included in this study.

### Sample size determination and sampling procedure

The sample size was calculated by using double population proportion formula indicated below with the following assumptions:

$$\frac{{{\text{n}} = \, \left[ {{\text{z}}\alpha /{2}\sqrt {\left( {{1} + {1}/{\text{r}}} \right)} + {\text{z}}\beta \sqrt {{\text{p}}_{{1}} \left( {_{{1}} - {\text{p}}_{{1}} } \right)} + {\text{p}}_{{2}} \left( {{1} - {\text{p2}}} \right)/{\text{r}}} \right]^{{2}} }}{{\left( {{\text{p}}_{{1}} - {\text{p}}_{{2}} } \right)^{{2}} }}$$

where; n represents the actual sample size, z α/2 is probability of committing type 1 error at 0.05 level of significance to be (± 1.96), zß is probability of rejecting a true difference is 20%, r is ratio of cases in exposed (n_1_) and unexposed group (n_2_) equals 1, p_1_: proportion of DEBs among adolescent with diabetes whose body mass index for age (BAZ) is ≥ + 1SD (overweight) to be 47.5%, p2: proportion of DEBs among adolescents with diabetes whose BAZ is ≥ − 2SD to < + 1SD (normal weight) to be 32.2%, and p_1_–p_2_ to be minimum meaningful difference in proportion between exposed and unexposed groups yielding a total sample size of 395.

In order to reach the study participants, first, a list of public hospitals in Addis Ababa was obtained and five randomly selected out of 12 public hospitals were included in the study. Secondly, study participants who were on follow up for at least one year in each selected hospital were identified by using baseline data that were obtained from diabetes follow-up registration book. Based on the total number of adolescents with diabetes available in each hospital, the computed sample size was allocated to each study hospital proportional to the number of adolescents with diabetes. Using the sampling frame, table of random numbers was generated using computer to select the study participants. Based on the number of adolescents with diabetes in each hospital, the number was assigned on the table from 1 to 3 digit numbers, and then the sample was randomly picked from the table until the required sample size was reached for each hospital. All T1-DM patients with psychological problem such as any mental retardation and autism were excluded from the study.

### Measurements and data quality assurance

Data were collected using a pretested and structured questionnaire. The questionnaire was first translated from English to Amharic and back-translated to English to maintain its consistency by linguistic experts in each field. The questionnaire consisted of questions related to socio- demographic characteristics, diabetes eating problem survey-revised (DEPS-R), body satisfaction scale, and diabetes complication. Moreover, patients’ cards were reviewed to know the type of diabetes and treatment status. The questionnaire was prepared in paper format and self-administered to the study participants after the consultation time in diabetes clinics.

Training was given for two data collectors and supervisors on objectives of study, confidentiality, technique of data collection including anthropometric measurement prior to data collection. The pre-test was conducted on 5% of the sample size in order to check for the suitability of the questions before the actual task of data collection. The questions used to measure DEBs (primary outcome of the study) and BSD (secondary outcome of the study) were cross-checked making use of the local version of the items. Accordingly, focus group discussions (FGDs) were conducted with similar study population in another setting to check for face validity of the questions. The internal consistency of DEPS-R was determined and revealed a good homogeneity of the scale items with a Cronbach’s Alpha equals 0.86 [36]. The results of the pre-test were analysed and incorporated in the final version of the questionnaire to make it more understandable in the field.

Data collection procedure was facilitated by two BSc nurses who were not working at the diabetes center during data collection time and the data collection process was supervised by a person holding a master’s degree in public health nutrition.

The DEBs were measured using DEPS-R, a diabetes-specific measure of DEBs, which is composed of 16 items on a 6-point Likert scale, ranging from 0 to 5, in relation to frequency of the behavior (0 = never; 1 = rarely; 2 = sometimes; 3 = often; 4 = usually; 5 = always) and can be completed in less than 10 min. Currently, DEPS-R tool is widely used by international researchers to identify high scores of DEBs in adolescents with T1-DM and its relationship with poorer glycemic control and psychological problems related to body image [37].

DEBs among adolescents with diabetes were defined as the presence of any of maladaptive behaviour in the past month preceding the survey which included eating excess amount of food in short time period; self-induced vomiting for weight control; the use of diuretics, laxatives, or insulin omission for weight control; or intense and excessive exercise for weight control (defined as > 30 min/day, predominantly for weight control, and not for fitness or leisure). Accordingly, a score of greater than 20 on DEPS-R score indicates the probability of having high risk for DEBs while a score ≤ 20 indicates lower risk of DEBs [36].

Body shape dissatisfaction was measured using a body part satisfaction scale which is composed of 8 items including face, upper torso, middle torso, lower torso, height, body weight, muscle tone, and overall appearance satisfaction. The scale was used to assess subjective appraisal of body image. Different parts of the body such as facial features, complexion, and hair, chest or breasts, shoulders, arms, waist, stomach, buttocks, hips, legs, and ankles alongside the height, weight and muscle tone of the individual were evaluated on 6 Likert’s scale: 1 = extremely dissatisfied; 2 = quite dissatisfied; 3 = somewhat dissatisfied; 4 = somewhat satisfied; 5 = quite satisfied, and 6 = extremely satisfied to appreciate body shape [38]. A higher score was considered to reflect more satisfaction with that aspect of the body while poor body shape satisfaction refers to negative subjective evaluations of one’s physical body, such as shape, weight, stomach and hips whereby body image satisfaction scale falls less than 16 [38].

Diabetes complications such as hypoglycaemia, diabetic ketoacidosis (DKA), microvascular complications that affect small blood vessels including retinopathy, nephropathy, and neuropathy were assessed by retrieving from registry of adolescents with diabetes with the support an endocrionologist or equivalent health care provider in the study facility [39].

Anthropometric measurements such as weight and height were done by the data collectors following the standard procedures. Adolescents were asked to wear light clothes and to be on bare foot before taking weight and height measurements. Weight was measured using standard digital balance with a precision of 0.1 kg whereas height was measured using a measuring board with a precision of 0.1 cm. The weight measurement scale was carefully handled and calibrated every day by placing 2 kg iron bar before the beginning of data collection for every eligible and data collectors checked whether the scales were adjusted at 0.00 reading before taking each weight measurement. To check the reliability, each participant was measured twice by each observer to assess intra-observer variability. Measurements done by each data collector were evaluated against measurements done by the criterion anthropometrist to minimize technical error of measurements (TEM) and thereby to ascertain validity of the measurements. The variability observed among data enumerators fell within the acceptable range of TEM for height and weight differences [40].

In this study, thinness refers to adolescents whose body mass index -for age (BAZ) is below − 2SD z score of WHO growth standard median reference among children and adolescent [41]. On the other hand, stunting refers to adolescent’s height for age Z-scores(HAZ) below − 2SD of the WHO growth median reference in children and adolescents [41].

Overweight refers to adolescents whose BAZ is between ≥  + 1SD and <  + 2SD WHO growth standard median reference among children and adolescent while obesity refers to having BAZ is ≥  + 2 SD above the WHO growth standard median reference in children and adolescent [41].

### Statistical analysis

The collected data were coded and entered on to Epidata version 4.4 and exported to SPSS version 24 for analysis. Descriptive statistics such as median and mean rank was used to describe the statistics of total score and sub-scales of DEPS-R`. Data were cleaned for outliers and corrected by transforming in to categorical variable if they were numeric and extreme values were omitted. The WHO Anthro Plus software was used to generate nutritional index, BAZ, for categorization. Non-parametric tests such as Mann–Whitney U-test and kruskal–Wallis test were used to evaluate the difference between median scores of independent variables such as BAZ and socio-economic status after checking for the assumptions of normality. Spearman correlation test and variance inflation factor were used to check for multicollinearity between predictor variables.

Variables with *p* value < 0.2 in bivariable logistic regression analyses were included into multivariable logistic regression model to assess the association between dependent and independent variables. Odd ratios alongside 95% confidence intervals (CIs) were estimated to measure the strength of association between variable of interest. In all cases, level of statistical significance was declared at p -value < 0.05.

## Results

### Socio-demographic characteristics of study participants

A total of 395 adolescents with diabetes participated in the study. Of all adolescents with diabetes, 52.2% were females while 47.8% were males. About 61% the study participants are in their early adolescence period (11–15 years). The median (± IQR) age of study participant was 15 (± 11 to 19) years. The majority of adolescents with diabetes were urban residents (93.4%) and 52.3% of them had attended secondary school followed by who attended the primary school (43.5%). Nevertheless, less than 3% of them attended some college /technical level school and above. Concerning paternal education status, nearly 28% of them had college graduate or above level of education while same proportion of mothers had attended secondary level of education. According to the 2022 Ethiopian average salary data, 67.1% of respondents are categorized in the middle income level (2251–8900) Ethiopian birr.

### Magnitude of disordered eating behaviors

Out of 395 participants, 86 (21.8%) females and 85 (21.5%) males had high risk for disordered eating behaviours. The overall magnitude of DEBs was 43.3%, [95%CI: (38.3%, 48.3%)]. There was significant difference in DEPS-R score between diabetes adolescents dissatisfied with their body shape (median (± IQR) = 23.5 (± 20.75), *p* = 0.001) and those satisfied with their body shape (median (± IQR) = 17 (± 16), *p* = 0.001).

The result also indicated that of diabetes adolescents who developed DEBs, the majority developed non-purging behaviour compared with purging behaviour. Most of them were alternating their meal followed by avoidance of checking their blood sugar (Table 1). However, there was no significant difference in DEPS-R score between males (median (± IQR) = 20 (± 15), *p* = 0.793) and females (median (± IQR) = 18 (± 19), *p* = 0.793).

**Table 1:** Purging and non-purging behaviours among adolescents with diabetes in the last 30 days prior to the survey, Addis Ababa, 2022 (n = 395)

	Yes n (%)	No n (%)
Purging behaviour		
Self -induced vomiting	8 (2.0)	387 (98.0)
Skipping insulin	30 (7.6)	365 (92.4)
Not taking enough insulin	43 (10.9)	352 (89.1)
Non -purging behaviour		
Skipping meal	45 (11.4)	350 (88.6)
Alternating eating	184 (46.6)	211 (53.4)
Eating more than usual	100 (25.3)	295 (74.7)
Avoiding checking blood sugar	105 (26.6)	290 (73.4)

### Body shape dissatisfaction and disordered eating behaviours

**Fig. 1:**
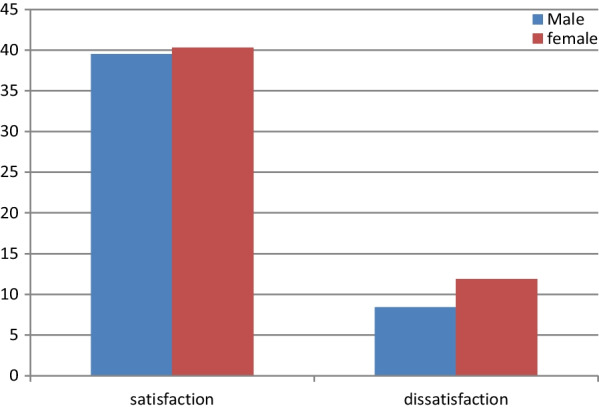
Distribution of body shape dissatisfaction by risk of disordered eating behaviours among diabetic adolescents, Addis Ababa, Ethiopia, 2022

The overall magnitude of body shape dissatisfaction among adolescents with diabetes was 20.3%, 95% CI: (16.4%, 24.6%)]. Nearly, twelve percent (11.9%) of female adolescents with diabetes were dissatisfied by their body shape while 8.4% of male adolescents with diabetes were dissatisfied by their body shape (Fig. 1).

Moreover, 38.2%, [95% CI: (33%, 43%) of diabetes adolescents were dissatisfied with their weight of which 22.3% of diabetes adolescents were dissatisfied with their weight and positive for DEBs and the rest 15.9% of them were dissatisfied with their weight and negative for DEBs respectively.

### Relationship of DEPS-R score with body mass index for age (zBAZ) and average income of diabetes adolescents

Kruskal Wallis test revealed that statistically significant difference in DEPS-R score across BAZ, X^2^ = 23.8, *p* < 0.001). DEPS-R scores were higher among overweight diabetes adolescents (median ± IQR): 27 (± 25) compared with diabetes adolescents who had low weight (thin) (median ± IQR):15 (± 13) and normal weight (median ± IQR):18 (± 15) respectively. Similarly, DEPS-R scores were higher among diabetes adolescents who had highest income (median ± IQR): 30 (± 23.25) compared with adolescents who had other income categories (Table 2).

**Table 2:** Significance difference between BMI-for-age (BAZ) and average income related with DEPS-R score, Addis Ababa, Ethiopia, 2022 (n = 395)

Variable	DEPS-R score (median)	*p* value	Kruskal–Wallis test	Mean rank
BAZ (total)	19	< 0.001	23.8	
Thin	15 (± 13)			127
Normal weight	18 (± 15)			189
Overweight	27 (± 25)			243
Average income (total)	19	0.004	13.15	
Low	15 (± 11)			154
Medium	19 (± 17)			197
High	20 (± 18)			225
Highest	30 (± 23.25)			246

### Factors associated with disordered eating behaviours among adolescents with diabetes

In multivariable logistic regression analysis, body shape dissatisfaction, family history of diabetes mellitus, late adolescence period, having diabetic complication, and being overweight were significantly associated with DEBs. Accordingly, the odds of DEBs were 2.2 times [(AOR = 2.21, 95% CI: (1.28, 3.82)] higher among adolescents who were dissatisfied with their body shape compared to their counterparts. Adolescents who had family history of DM were 1.6 times [(AOR = 1.59, 95% CI: (1.03, 2.47)] more likely to have DEBs compared with those who did not have family history of DM. Adolescents with diabetes who are in late adolescence period had 2 times [(AOR = 2.10, 95% CI: (1.33, 3.34)] higher odds of having DEBs compared with adolescents in their early adolescence period. Adolescents having diabetic complications were 2.3 times [(AOR = 2.32, 95% CI (1.43, 3.75)] more likely to engage in DEBs compared with their counterparts. Moreover, the odds of DEBS were 2 times [AOR = 2.25, 95% CI (1.32, 3.82)] higher among overweight study participants compared with those having normal nutritional status (Table 3).

**Table 3:** Factors associated with DEBs among adolescents with diabetes on follow-up in selected public hospitals, Addis Ababa, Ethiopia, 2022

Variables	DEBs		COR (95% CI)	AOR (95% CI)
	Yes n (%)	No n (%)		
Satisfaction with body shape				
Yes	124 (39.37)	191 (60.63)	1	1
No	47 (58.75)	33 (41.25)	2.19 (1.33, 3.61)**	2.21 (1.28, 3.82)***
Parents’ average monthly income				
Low	15 (29.4%)	36 (70.6%)	1	1
Middle	116 (43.8%)	149 (56.2%)	1.87 (0.98, 3.58)	1.21 (0.600 2.44)
High	40 (49.3%)	39 (50.7%)	2.45 (1.17, 5.19)*	1.57 (0.69, 3.53)
Family history of DM				
No	78 (38.0%)	127 (62.0%)	1	1
Yes	93 (48.9%)	97 (51.1%)	1.56 (1.05, 2.33)*	1.59 (1.03, 2.47)*
Adolescence period				
Early adolescence	90 (37.34)	151 (62.66)	1	1
Late adolescence	81 (52.60)	73 (43.40)	1.86 (1.24, 2.81)**	2.10 (1.33, 3.34)**
History of previous admission				
No	20 (30.8%)	45 (69.2%)	1	1
Yes	151 (45.8%)	179 (54.2%)	1.89 (1.07, 3.35)*	1.41 (0.75, 2.64)
Diabetes complication				
No	40 (28.99)	98 (71.01)	1	1
Yes	131 (50.97)	126 (49.03)	2.55 (1.64, 3.96)**	2.32 (1.43, 3.75)**
Nutritional status of adolescents				
Normal weight	114 (40.14)	170 (59.56)	1	1
Thin	3 (15)	17 (85%)	0.26 (0.08, 0.92)*	0.37 (0.10, 1.36)
Overweight	54 (59.34)	37 (40.66)	2.18 (1.35, 3.52)**	2.25 (1.32, 3.82)**

*p < 0.05, **p < 005, ***p < 0.0001

## Discussion

This study aimed to examine the magnitude of DEBS and its association with BSD among adolescents with diabetes. The magnitude of DEBs in this study was 43.3%. DEBs was significantly associated with BSD, family history of diabetes mellitus, late adolescence period, having diabetic complication, and being overweight. On the other hand, the magnitude of BSD among the study subjects was 20.3%.

In this study, the magnitude of DEBs was 43.3% among adolescents with diabetes in the past 30 days. This suggests that this high magnitude of DEBs may be a clue that the western view of leanness as a sign of beauty and attractiveness is no longer limited to those countries. In low and middle income countries, the prevalence of DEBs among adolescents is also escalating ranging from 7.4%-39.3% of which girls are more likely to get engaged in such practices than boys [42, 43]. The possible explanation for this difference may be attributed to use of various cut off points, study populations, and different reference period across the former studies since some studies used 30 days reference period and others used one year period. The observed magnitude in this study is different from the results of other better off countries, 21% in Norway and 68% in Turkey [10, 44]. The reason may be attributed to the difference in the measuring tools used to measure the outcome, for instance, in Turkey the scholars used more generic tool (eating attitude test), which often overestimates the prevalence of DEBs in population with diabetes.

Evidence from studies conducted in Egypt showed that the magnitude of DEBs among type-1 diabetes is 34.7% [16] and 29.4% Tunisia [15]. These findings are lower than the result of the present study. The possible explanation for this difference may be due to the fact that our study used small sample size and different study subjects in relation their age range.

Few studies from Ethiopia reported the prevalence of disordered eating attitude and unhealthy weight control behaviours. Accordingly, the prevalence of disordered eating attitude among adolescents was 8.6% [34] while the level of unhealthy weight control behaviors was 30.7% [33] respectively. The reason behind the difference between the present study and former studies may be the use of different measuring tools (eating attitude test − 26, which measures eating attitude towards dieting and bulimia), difference in reference periods and study population. The present study used diabetes eating problem survey (DEPS-R) questionnaires among adolescents with diabetes. It is believed that the result of this study would serve as the baseline indicating the need for further future research using context specific validated tool.

The magnitude of BSD among the study subjects was 20.3%. Nearly 12% of female adolescents with diabetes are dissatisfied with their body shape while 8.3% of male adolescents with diabetes are dissatisfied with their body shape. This implies that females are more concerned with their body shape than males. Our finding is consistent with a national survey conducted among Brazilian adolescents which revealed 30% of body weight dissatisfaction whereby girls had the highest percentage of dissatisfaction [45]. In a cross-sectional study conducted in a Municipality of Spain, 40% of adolescents were dissatisfied with their own body weight [46].

Moreover, BSD is strongly associated with DEBs in this study. The odds of DEBs are two times higher among adolescents with diabetes compared with their counterparts. This finding is consistent with previous studies conducted in different settings of the world [5, 6, 14, 25, 29, 38, 47]. This implies that adolescents who are dissatisfied with their body shape are often engaged in DEBs in fear of having poor body image which they feel can be regulated through either weight loss or skipping regular meals.

The odds of having DEBs are nearly two times higher among adolescents from families who had history of diabetes mellitus. This finding goes with evidence from previous narrative review where empirical evidence of family relationships in adolescents’ eating disorders confirms the relevance of relational aspects in the development and maintenance of DEBs [48]. In the same line, the family eating environment and diabetes family conflict may represent important factors for disordered eating risk in adolescents with T1-DM [49]. Nevertheless, no direct evidence was reported to reveal an association between family history of DM and adolescents’ DEBs which requires further investigation.

Being in late adolescence period is significantly associated with engagement in DEBs. Adolescents in their late adolescence period are nearly twice at risk of getting engaged in DEBs compared with their counterparts. This finding is in congruence with study from Norway which reported the magnitude of DEBs to be 41.2% among adolescents aged 17–19 years, 19.8% among adolescents aged 14–16 years and 3.6% among those aged 11–13 years [10]. This may be due to the fact that the risk of DEBs increases as age increases due to hormonal and musculoskeletal related changes resulting in change in body shape and life style.

Having diabetic complication is turned significant to have an association with DEBs. The odds of DEBs are two times higher among study subjects who developed diabetes complication compared with their counterparts. This finding is in an agreement with findings from studies conducted in Spain and Egypt [39, 46]and systematic review [50]. This implies that young population with diabetes (PwD) who practice DEBs are easily prone to complications related with T1-DM varying from acute complication to severe stage or chronic complications.

Nutritional status of adolescents was measured and associated with DEBs in this study. The odds of engagement in DEBs are two times higher among overweight adolescents compared with normally nourished ones. This finding is in accordance with other studies which revealed that overweight was found to be a risk for the development of DEBs [5, 8, 22, 27, 36, 51–56]. This implies that overweight adolescents may worry about their physical appearance compared with undernourished or normally nourished ones and exercise altering their eating behaviour.

In contrast to the former literatures, in this study, socio- demographic and economic characteristics of adolescents such as income [5, 55]; gender [7, 9, 22, 27, 53, 56]; and age [9] are not significantly associated with DEBs. Evidence from these literatures indicated that adolescents from better-off families are more likely to be exposed to social influence and easy access to various social media which can lead to body weight misperception, body part dissatisfaction, and depressive symptoms. It has also been argued that adolescents from wealthy homes are more likely to get accurate information regarding body weight awareness and healthy weight management behaviours because they have access to this information through numerous pathways.

This study may have the following limitations:- Firstly, the study was conducted only among adolescents with diabetes which might not prove its generalizability to the general population. Secondly, there may be measurement error while measuring the outcome variable of interest, DEBS, using the existing tool which is not yet validated in the study setting though widely used across the globe for the same purpose. Thirdly, there may be limitation which may be attributed to lack of temporal relationship, for instance, it might not be possible to describe whether a change in BMI is preceded by engagement in DEBs and vice-versa. Fourthly, TEM might lead to misclassification of the nutritional status of the study participants, although all necessary actions were taken in standardizing anthropometric measurements before and during anthropometric measurements to minimize measurement errors. Lastly, but not least, the outcome variable was measured using DEPS-R questionnaire which has not yet been validated in study setting. However, efforts have been made to make the tool understandable by the study participants before its administration. Accordingly, focus group discussions were conducted in other hospitals with T1-DM adolescent patients with the aim of assessing the face validity of the tool such as clarity of wording and the likelihood that the study population are able to respond to the questions as appropriate as possible. The discussion was held with the local language, Amharic, and some items with difficulty to understand were paraphrased based on their suggestions for final data collection. The researchers believe that this approach could have minimized biases that could have been introduced otherwise. It is also promising that the tool has been contemporarily validated for this purpose and is currently in use by wide group of international researchers as a standard tool for the same purpose [36, 37].

## Conclusions

The magnitude of DEB was high among adolescents with diabetes in selected public hospitals, Addis Ababa. In adjusted multivariable analysis, BSD, family history of diabetes mellitus, being in late adolescence period, having diabetic complication, and nutritional status of adolescents were significantly associated with DEBs. Therefore, preventive interventions need to be designed by all relevant actors working on health promotion of young population to address factors influencing disordered eating behaviours among adolescent population with diabetes.

## Data Availability

Data can be reachable by sending an email to the corresponding author. If needed, the results and database can be uploaded when need arises.

## Abbreviations

AAU: Addis Ababa University
AOR: Adjusted odds ratios
BAZ: Body Mass Index for age
BSD: Body shape dissatisfaction
CI: Confidence interval
COR: Crude odds ratio
DEBs: Disordered eating behaviours
DEPS-R: Diabetes Eating Problem Survey-Revised
HAZ: Height for age Z-score
IRB: Institutional review board
IQR: Inter quartile range
DKA: Diabetic ketoacidosis
LDL: Low density lipoprotein
PwD: Population with diabetes
SD: Standard deviation
T1-DM: Type 1 diabetes mellitus
T2-DM: Type 2 diabetes mellitus
zBMI: Body Mass Index Z-score
WHO: World Health Organization
